# Balancing personal maintenance with parental investment in a chick-rearing seabird: physiological indicators change with foraging conditions

**DOI:** 10.1093/conphys/cox055

**Published:** 2017-09-26

**Authors:** Anne E Storey, Morag G Ryan, Michelle G Fitzsimmons, Amy-Lee Kouwenberg, Linda S Takahashi, Gregory J Robertson, Sabina I Wilhelm, Donald W McKay, Gene R Herzberg, Frances K Mowbray, Luke MacMillan, Carolyn J Walsh

**Affiliations:** 1 Department of Psychology, Memorial University, St. John’s, Newfoundland and Labrador, Canada A1B 3×9; 2 Department of Biology, Memorial University, St. John’s, Newfoundland and Labrador, Canada A1B 3×9; 3 Cognitive and Behavioural Ecology Graduate Program, Memorial University, St. John’s, Newfoundland and Labrador, Canada A1B 3×9; 4 Environment and Climate Change Canada, 6 Bruce St., Mount Pearl, Newfoundland and Labrador, Canada A1N 4T3; 5 Faculty of Medicine, Memorial University, St. John’s, Newfoundland and Labrador, Canada A1B 3V6; 6 Department of Biochemistry, Memorial University, St. John’s, Newfoundland and Labrador, Canada A1B 3×9; 7 Fisheries and Oceans Canada, P.O. Box 5667, St. John’s, Newfoundland and Labrador, Canada A1C 5×1

**Keywords:** beta-hydroxybutyrate, body mass, capelin, common murres, corticosterone, foraging conditions, haematocrit, physiological indicators

## Abstract

Seabird parents use a conservative breeding strategy that favours long-term survival over intensive parental investment, particularly under harsh conditions. Here, we examine whether variation in several physiological indicators reflects the balance between parental investment and survival in common murres (*Uria aalge*) under a wide range of foraging conditions. Blood samples were taken from adults during mid-chick rearing from 2007 to 2014 and analysed for corticosterone (CORT, stress hormone), beta-hydroxybutyrate (BUTY, lipid metabolism reflecting ongoing mass loss), and haematocrit (reflecting blood oxygen capacity). These measures, plus body mass, were related to three levels of food availability (good, intermediate, and poor years) for capelin, the main forage fish for murres in this colony. Adult body mass and chick-feeding rates were higher in good years than in poor years and heavier murres were more likely to fledge a chick than lighter birds. Contrary to prediction, BUTY levels were higher in good years than in intermediate and poor years. Murres lose body mass just after their chicks hatch and these results for BUTY suggest that mass loss may be delayed in good years. CORT levels were higher in intermediate years than in good or poor years. Higher CORT levels in intermediate years may reflect the necessity of increasing foraging effort, whereas extra effort is not needed in good years and it is unlikely to increase foraging success in poor years. Haematocrit levels were higher in poor years than in good years, a difference that may reflect either their poorer condition or increased diving requirements when food is less available. Our long-term data set provided insight into how decisions about resource allocation under different foraging conditions are relating to physiological indicators, a relationship that is relevant to understanding how seabirds may respond to changes in marine ecosystems as ocean temperatures continue to rise.

## Introduction

Parents must balance the costs of care for offspring with possible negative consequences for their future survival and reproduction. These trade-offs are key to the prudent parent model (Drent and Daan, 1980; Bókony *et al.*, 2009), as life history parameters determine how much can be invested in current offspring without compromising future reproductive success. Long-lived organisms may need to reduce care or abandon offspring since their lifetime reproductive success depends more on their survival than on success in any single reproductive attempt (Stearns, 1989; Satterthwaite *et al.*, 2010).

Several factors make reproductive costs for murres (*Uria* spp.) particularly high, even among long-lived seabirds. Due to their diving-adapted wings, murres have elevated flight costs associated with extremely high wing loading (Ainley *et al.*, 2002; Elliott *et al.*, 2013). Murre parents take turns foraging and remaining on the cliff with their single chick and this constant chick attendance reduces the time available for foraging. Under poor foraging conditions, common murres *U. aalge* decrease their chick provisioning rates (Harris and Wanless, 1988; Burger and Piatt, 1990; Harding *et al.*, 2007; Wilhelm *et al.*, 2008), which is consistent with predictions for low brood value species (Bókony *et al.*, 2009; Breuner, 2010).

Various physiological indicators have been used to assess how organisms respond to environmental variation. These include mass and/or body condition indices, lipid metabolites, blood oxygen capacity (haematocrit) and corticosterone (CORT) levels. Mass and related body condition indices are the least intrusive measures but it remains unclear whether decreases reflect differences in lipid storage or muscle loss in all species (Jacobs *et al.*, 2011, 2012). Changes in body mass in alcid seabirds primarily reflect changes in lipid storage (Niizuma *et al.*, 2002; Elliott *et al.*, 2008; Jacobs *et al.*, 2011, 2012), so measures of mass or mass loss during chick rearing can be used as indicators of energy allocation to self and/or offspring (e.g. Gaston and Hipfner, 2006a; Piatt *et al.*, 2007; Rector *et al.*, 2012).

Adult murres lose mass between incubation and chick rearing (Croll *et al.*, 1991; Gaston and Hipfner, 2006b; Jacobs *et al.*, 2011). The rapid onset of mass loss at hatching suggests that it is ‘programmed’ (Croll *et al.*, 1991; Jones, 1994), to reduce the costs of diving (Elliott *et al.*, 2008) and/or flying back and forth to the colony (e.g. Blem, 1976; Croll *et al.*, 1991). Under poor foraging conditions, murres lose more mass (Gaston and Hipfner, 2006b) and they lose it earlier in chick rearing (Wilhelm, 2004). Beta-hydroxybutyrate (BUTY), the major ketone body in birds, is produced when lipid stores are metabolized and mass decreases (Totzke *et al.*, 1999). BUTY concentrations are more associated with mass loss (vs. gain) than other lipid metabolites (Cerasale and Guglielmo, 2006; Seewagen *et al.*, 2011). BUTY levels rise when calorie-dense lipid stores are metabolized and this is accompanied by gradual mass loss (Totzke *et al.* 1999). Elevated BUTY levels are associated with mass loss involving lipid metabolism in early and middle stages of fasting in emperor *Aptenodytes forsteri* and king penguins *Aptenodytes patagonicus* (Cherel and Le Mayo, 1985, 1988; Robin *et al.*1988; Groscolas and Robin, 2001). It may be advantageous for murres to delay or minimize mass loss, and thus, variation in BUTY levels may provide information about the timing of mass loss under different foraging conditions.

Haematocrit levels, the ratio of red blood cell (RBC) volume to total RBC and plasma volume, are often positively related to overall good body condition and health (reviewed in Fair *et al.*, 2007). Fair *et al.* (2007) caution against using haematocrit levels as the only measure of good condition as elevated levels may also result from the organism being compromised or physiologically less efficient (e.g. higher haematocrits in humans who smoke, Sagone *et al.*, 1973; American kestrels *Falco sparverius* with blood parasites, Dawson and Bortolotti, 1997; Adélie penguins *Pygoscelis adeliae* with long fasts, Vleck *et al.*, 2000). In contrast, higher haematocrit levels can also allow greater blood oxygenation (as in diving seals, Thornton and Hochachka, 2004) and higher levels are associated with the deeper dives and larger body mass in diving mammals (Hedrick and Duffield, 1991), and longer dives in some seabird species (Elliot *et al.*, 2010; Crossin *et al.*, 2015).

CORT levels are thought to reflect stresses associated with current and recent challenges, particularly those involved in regulating metabolism, including energy mobilization, acquisition and storage (e.g. Kitaysky *et al.*, 2007; Doody *et al.*, 2008; Barrett *et al.*, 2015). Higher CORT levels are associated with reduced food availability (reviewed in Breuner, 2010) and are often associated with decreased body condition and reproductive investment (CORT-fitness hypothesis, Bonier *et al.*, 2009). Alternatively, elevated CORT has been observed to increase foraging effort and energy storage (CORT-adaptation hypothesis, Bonier *et al.*, 2009). Results vary as to whether CORT-induced increases in foraging effort benefit parents (e.g. Almasi *et al.*, 2008; Horton *et al.*, 2009), chicks (Bonier *et al.*, 2011) or both (e.g. Angelier *et al.*, 2007, 2008; Doody *et al.*, 2008; Crossin *et al.*, 2012). Parents in good condition may be able to increase CORT and maintain chick-feeding rates while parents in poor condition cannot (Angelier *et al.*, 2007, 2008; Doody *et al.*, 2008). Offspring may benefit only when increases in their parents’ CORT levels are moderate, but not when elevations are large and/or prolonged (Breuner, 2010).

Prey fluctuations, which are increasingly common for nesting seabirds in the North Atlantic, are associated with long-term changes in ice conditions and water temperature (Buren *et al.*, 2014). Physiological indicators that are potentially linked to this variation in food availability are: body mass which reflects lipid stores; BUTY as a measure of mass loss; CORT as a metabolic regulator involved in energy balance and haematocrit as either a general body condition or health indicator or a measure of diving effort. Long-term studies are the best way to understand how environmental variation affects physiology (Williams *et al.*, 2008; Crespi *et al.*, 2013). Further, we try to determine what physiological processes can be used to assess breeding costs (Golet *et al.*, 2004; Williams and Fowler, 2015) and whether individual differences in exercise capacity, as measured by these physiological markers, plays a roles in variation in breeding success (as suggested in Yap *et al.*, 2017). Here we compare these physiological measures in common murres across eight breeding seasons in which foraging conditions vary, to determine which measures are related to murres investing conservatively in offspring, particularly under poor conditions, i.e. acting as prudent parents. Alternatively, high quality murres may be able to afford the cost of elevated physiological indicators (i.e. may not respond conservatively) without compromising future reproductive success. If parents act prudently, we would expect chick-feeding rates and reproductive success to vary positively with the adult indicators associated with good body condition. We predict that when fish are less available, parents will have lower body mass, higher BUTY levels (if mass loss is ongoing at the time of sampling), higher CORT levels and low chick-feeding rates. Our previous results for murres supports the CORT-adaptation hypothesis in that murres with higher CORT in a poor year lost less mass and had higher chick- feeding rates (Doody *et al.*, 2008). Here we will test whether our previous results for CORT extend to a wider range of foraging conditions that may further affect the balance between parental investment and personal maintenance. It is difficult to make a directional prediction for haematocrit values: there may be a positive relationship if higher haematocrit levels are associated with the better body condition (e.g. many references in Fair *et al.*, 2007) in good foraging years. In contrast, haematocrits may be elevated if increased diving requirements are associated with poor foraging conditions, resulting in a negative relationship.

## Materials and methods

### Study site and sampling

Adult common murres (*N* = 143) were captured from the same plot in a colony on Gull Island (47°16´N, 52°46´W), Witless Bay Ecological Reserve, Newfoundland and Labrador, Canada over 8 breeding seasons. Birds were caught on mornings between July 8th and 19th from 2007 to 2014 (*N* = 10–28 birds per year) when chicks were, on average, 10 days old (in the approximately 3-week pre-fledge period). Yearly sample sizes were small because extending capture duration can be disruptive when chicks start to move from their nest sites. Due to the relatively small sample sizes each year, we grouped the eight years of data into three year types so that we could reach general conclusions about the effects of large annual differences in foraging conditions (details on year assignments to follow). Twenty birds were captured more than once (for a total of 31 recaptures). Analyses were done on the entire sample (*N* = 143) or just the first captures (*N* = 112) with very similar results. The latter are reported.

Each bird was captured using a 7 m noosepole and placed head first into a cloth bag. The bird and bag were quickly removed to a nearby site behind the cliff, out of view of the murre colony. A 23-gauge × 1.9-cm winged-infusion needle (Terumo Surflo®, Fisher, Ottawa, ON) attached to a 3 ml syringe barrel (Luer-Lok™ Tip, Sigma-Aldrich, Oakville, ON) was used to collect approximately 2.5 ml of blood from the brachial vein within 3 minutes of capture. Blood drops were dispensed onto blood spot filter paper cards (Whatman, GE Healthcare Life Sciences) and were dried for 24 hours. Birds were weighed (Pesola scale, ITM instruments, Montreal, QC) and banded (plastic colour and Canadian Wildlife Service bands).

The remaining blood was transferred to non-heparinized blood collection tubes and from 2010 to 2014 to heparinized capillary tubes (Fisher Scientific, Ottawa, ON) that were sealed at one end using Critoseal® (Fisher Scientific, Ottawa, ON). Capillary and blood collection tubes were spun for 10 min at 2200 g using a Galaxy 7D VWR centrifuge (VWR, Edmonton AB). The haematocrit percentage was calculated for each capillary tube: the length of the RBC section divided by the length of total sample. The plasma from each collection tube was extracted and placed in a new vial before freezing at −20 °C.

### Beta-hydroxybutyrate assay

Plasma samples were analysed for BUTY concentration using a microplate spectophotometer (Biotech Powerwave XS, Fisher Scientific, Nipean ON) and a kinetic endpoint assay (kit E0907979, R-Biopharm, Marshall, MI). We used procedures described in Guglielmo *et al.* (2002) and Guglielmo *et al.* (2005). BUTY samples were available for all years except 2013. Interplate variation for high standard values was 10.8% and for low standard values was 4.2%. Samples were run in duplicate and the mean CV was 8.3%.

### Corticosterone assay

Blood spot and plasma total CORT concentrations were measured with COAT-A-COUNT Rat CORT ^125^I radioimmunoassay kits (Cat. # TKRC1, InterMedico, Markham, Ontario), using the kit’s normal procedures for plasma serum, modified for blood spots (detailed and validated in Doody *et al.*, 2008; Rector *et al.*, 2012). This assay has a 2.9% cross-reactivity with 11-deoxycorticosterone and <1 % cross-reactivity with other steroids, including progesterone. Blood spots were used because of their greater convenience in the field and because they retain higher hormone levels than long-stored plasma samples (Rector *et al.*, 2012). To standardize CORT levels between yearly assays, pooled samples were also prepared: two sets of two 3.2 mm punches from each of six cards were cut, corresponding to samples from six different common murres, to match the number of punches in replicates for the samples (as in Doody *et al.*, 2008; Rector *et al.*, 2012; Fitzsimmons *et al.*, 2017). As in these previous studies, assay values were adjusted relative to the pooled sample to allow between-year comparisons (no adjustment for 2007, 2008, 2010 and 2012, reduced by 14% in 2013 and 2014; increased by 13% in 2011 and 21% in 2009). These adjustments made our analyses more conservative by decreasing the assay-induced differences among years (e.g. 2009 had both the lowest mean CORT levels for samples and the lowest pooled murre values). Intra-assay coefficients of variation (%CV) of low and high blood spot CORT values were between 4.5–8.1% and 4.2–6.3%, respectively. All bloodspot values (mean, 18.1 ng/ml) were within the straight-line portion of the standard curve, well above the assay’s lower limit of sensitivity (5 ng/ml). Plasma assays from a sample of the same birds allowed us to convert blood spot samples to more familiar plasma values. The conversion formula was Plasma = (blood spot +7.89)/6.24. CORT and BUTY values were log-transformed to reduce skew.

As in our previous research (Doody *et al.*, 2008), there was no significant relationship between capture order and CORT levels, indicating that our presence adjacent to the colony (but out of sight of the birds except for the actual capture) did not result in increasing CORT levels during the capture sessions.

### Sex determination

DNA was extracted from blood spot cards using DNeasy Blood & Tissue Kits (QIAGEN, Toronto, ON) and individuals were sexed using a CHD (chromodomain helicase DNA)-based molecular method (Fridolfsson and Ellegren, 1999). Highly conserved primers (2550 F and 2718 R) were used. Females were identified by two fragments (CHD1W and CHD1Z); males by one fragment (CHD1Z), following polymerase chain reactions and agarose electrophoresis.

### Assessment of yearly differences in capelin availability

Although no estimates of local capelin abundance are available for the chick-rearing period, indices of abundance derived from acoustic surveys two months earlier (May), coupled with observations of capelin spawning activities and egg deposition during chick rearing (Fisheries and Oceans Canada, DFO), indicate that there is considerable interannual variability in both the biomass and timing of the inshore spawning migration of capelin (*Mallotus villosus*). This species comprised at least 90% of prey provisioned to chicks at the same colony from 2007–2010 (Regular *et al.*, 2014), so indices of capelin availability should reflect general foraging conditions for chick-rearing murres. Drivers of the variability in abundance and timing of spawning include environmental factors affecting both cohort strength during the larval stage (e.g. water temperature) and the timing of spawning (size and age of spawners; Nakashima, 1996; Carscadden *et al.*, 1997). Spring ice coverage and the timing of ice retreat may also indirectly impact capelin spawning through effects on the phytoplankton bloom consumed by copepods, the main prey of capelin (Buren *et al.*, 2014).

We used DFO data from Bellevue Beach, Trinity Bay, 80 km from Witless Bay and the closest location to our study site with comprehensive spawning data. These data included date of peak spawning (Column 1, Table 1) and the degree to which the spawning duration overlapped with chick rearing (Column 2, Table 1, see also Fig. 1 in Regular *et al.*, 2014 for 2007–2010). DFO-sponsored capelin daily diaries, kept by a fisherman at each of Ferryland near Gull Island, Witless Bay (47°1′0″N, 52°53′0″W, 2007–2014) and Bellevue Beach (47°38′2″N, 53°45′59″W, 2009–2014) in Trinity Bay (Nakashima, personal communication) were used to provide information on short-term changes in capelin spawning (Column 2, Table 1). The assessment categories for the diaries included daily observations of light, medium or heavy spawning, as well as sightings of small, medium or large capelin schools. These data have been used to assess capelin availability for alcid seabirds in Witless Bay in several publications (Doody *et al.*, 2008; Wilhelm *et al.*, 2008; Rector *et al.*, 2012; Regular *et al.*, 2014; Fitzsimmons *et al.*, 2017).

**Table 1:** Assessment of year categories (poor, intermediate, good) is based on peak and duration of spawning (DFO spawning data, including daily diaries) and abundance indices from spring acoustic surveys. Chicks hatch in late June/early July and fledge in late July

Year	1. Peak spawning	2. Complete overlap with chick rearing?	3. Abundance indices from Acoustic Survey data^a^	4. Year Category assigned for Gull Island, Witless Bay (rationale including diary comments)
2007	Mid July	Yes	Low	Intermediate (medium stock, spawning overlapped with chick rearing)
2008	Mid July	Yes	Low	Intermediate (medium stock, spawning overlapped with chick rearing)
2009	Early August	No spawning for most of chick rearing	Low	Poor (medium stock, latest spawning)
2010	Mid July	Yes	Lowest	Poor (lowest stock)
2011	Early July	Spawning low after mid-chick rearing; (diary: no high days after July 15)	Medium	Intermediate (little spawning after mid-chick rearing)
2012	Mid July	Yes	Medium	Good (good stock, spawning occurring throughout chick rearing)
2013	Early July	Spawning low after mid-chick rearing (diary: no high days from July 4 to 22	High	Intermediate (low spawning in mid-chick rearing)
2014	Mid July	Yes	High	Good (highest spring assessment, spawning occurred throughout chick rearing

^a^Lowest, 0.99 billion in 2010; Low, 10–20 billion; Medium, 50–70 billion; High, 100–360 billion.

**Figure 1:**
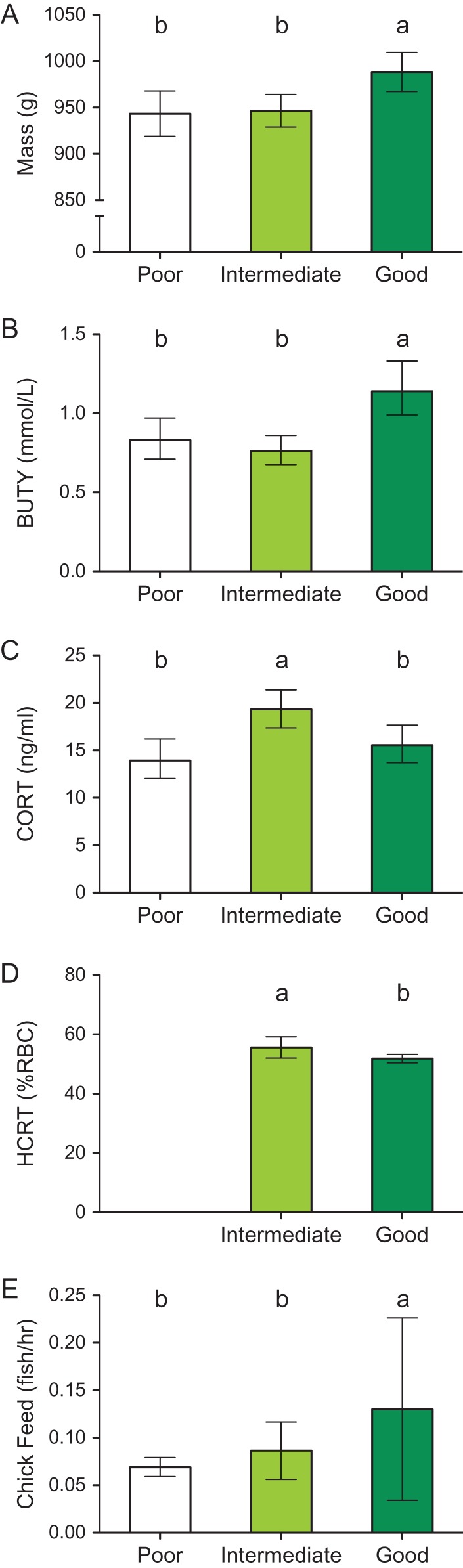
Mean and confidence intervals for poor intermediate and good years for (**A**) Mass, (**B**) BUTY levels, (**C**) CORT levels, (**D**) haematocrit (HCRT) and (**E**) chick-feeding rate. Significant differences are indicated by different letters above bars.

Our comparison spanned more years than the studies referenced above and so we were interested in adding any other information that might relate to overall capelin abundance. Indices of capelin abundance derived from May acoustic surveys for the near shore portion of Area 3 L (Atlantic Ocean) near Witless Bay were provided by DFO (Mowbray, 2014; DFO, 2015; Mowbray, unpublished data, Column 3, Table 1). Based on all these sources we categorized years as either poor, intermediate or good capelin availability (Column 4, Table 1). Good years had high capelin abundance and the timing of inshore spawning overlapped with chick rearing. Intermediate years had either high capelin abundance, but inshore timing did not completely overlap with chick rearing (2011, 2013), or moderate capelin abundance (2007, 2008). Poor years had low overall capelin abundance and/or a mismatch in the timing of murre chick rearing and inshore arrival of capelin. Capelin stocks were more abundant in the more recent years (2011–2014, two intermediate and two good years) than in the early years (2007–2010, two intermediate and two poor years).

Breeding success data for Witless Bay murre colonies (Regular *et al.*, 2014, plus data for additional years compiled by Environment and Climate Change Canada and our group) are consistent with the assessment of 2009 and 2010 as poor years. Fledging success averaged 35% in those two years compared to an average of 65% in the other years. Intermediate and good years did not differ in fledging success.

### Nesting success

Murres with chicks that survived to two weeks after hatching were defined as successful breeders since our previous data showed that almost all chicks that survive to this point go on to fledge (hereafter called nest success). For birds caught between 2007 and 2012, we recorded their nest success for the next three years and related the number of successful breeding attempts to the physiological indices in the year they were caught.

### Chick-feeding rates

Chick-feeding rates were available for three years: a poor year (2009), an intermediate year (2013) and a good year (2014). Data were obtained from watches of 6–15 hour durations (number of watches: 7 in 2009; 11 in 2013; 16 in 2014). Feedings were confirmed by video analysis for captured individuals. Murres bring a single fish to their chicks and the frequency of fish deliveries was converted to an hourly rate for each year.

### Statistical analyses

Multivariate analysis of variance (MANOVA) was used to test whether there were differences in mass, BUTY levels, and CORT levels in (a) the three year types (poor, intermediate and good; proxy for capelin availability) followed by LSD post hoc tests, (b) for successful and unsuccessful breeders, c) for males and females, and d) for early and late sampling within the mid-chick-rearing week (proxy for changes during chick rearing). Julian date was added as a covariate in the first three analyses, as some indicators changed with date, but there was no difference in the pattern of results with or without this covariate. The analysis for nest success was run initially on all first-captured birds and then, to exclude definite non-breeders, we included only murres with at least one successful breeding attempt. Results were the same in both cases and this latter analysis is reported. Haematocrit percentages were analysed separately because these data were not collected in all years. For haematocrit values, years were grouped as either good (2012 and 2014) or intermediate years (2011 and 2013) and analysed with independent *t*-tests. Other analyses were performed with one-way ANOVAs (e.g. comparisons of indicators for birds that varied in the number of successfully fledged chicks), independent *t*-tests (e.g. comparison of mass of birds captured in more than one year) and Pearson r correlations (e.g. relationship among indicators). CORT and BUTY levels were log-transformed to reduce skew. The pattern of results was the same for analyses with log and raw data and the former are reported. Nonparametric tests were used when homogeneity of variance assumptions were violated (e.g. chick-feeding data, Kruskal–Wallis test followed by Dunn–Bonferroni post hoc test). Means for CORT and BUTY levels were back transformed to have meaningful units for the text and figures. All means are presented with confidence intervals.

## Results

### Relationships among physiological variables

Adult mass was negatively correlated with haematocrit levels (Pearson’s *r* 40 = −0.39, *P* = 0.013). There were no other significant correlations among the variables in the analysis that included mass, BUTY, CORT and haematocrit.

### Effects of capelin availability

Mass, CORT levels and BUTY levels across capelin availability indices (overall MANOVA with time in chick rearing as a covariate, *F*6,152 = 7.49, *P* < 0.001, Wilks *λ* = 0.60, *η*^2^ = 0.23). Murres weighed significantly more in years with good capelin availability than in intermediate years (LSD post hoc test, *P* = 0.003) and poor years (*P* = 0.007, Fig. 1A). BUTY levels showed the same pattern as mass with higher levels in good years than in intermediate (*P* = 0.001) and poor years (*P* = 0.007, Fig. 1B). There were no differences in both mass and BUTY levels between poor and intermediate years. Murres had higher CORT levels in the intermediate capelin availability years than in both the good years (*P* = 0.039) and poor years (*P* = 0.043), with no detectable difference between the good and poor years (Fig. 1C). Haematocrit levels were lower in good years than in intermediate years (good, 51.2% ± 0.5; combined intermediate/poor, 54.6% ± 1.5, *t*61 = 2.3, *P* = 0.024, Fig. 1D).

Individual murres (*N* = 11) that were captured in two different years, and were successful breeders in both were significantly heavier in the year with better foraging conditions (better year, 971.3 ± 9.6 g; worse year, 951.7 ± 14.3 g, *t*10 = 2.93, *P* = 0.02). No other variables differed for birds caught more than once.

Chick-feeding rates varied significantly across foraging conditions (Kruskal–Wallis test, *P* = 0.034, *η*^2^ = 0.25). Chick-feeding rates were significantly higher in the good year (2014, mean rank = 19.6, 2.1 fish per day) than in the poor year (2009, mean rank = 9.5, fish 1.1 fish per day, Dunn-Bonferroni post hoc test, *P* = 0.023). Chick-feeding rates did not differ between good and intermediate years (2013, mean rank = 15.8, 1.44 fish per day) and there was a marginally higher chick-feeding rate in the intermediate year than in the poor one (*P* = 0.056, Fig. 1E).

### Nest success

Mass was the only significant variable in the analysis of differences between successful and unsuccessful murres (overall MANOVA with capture date as a covariate *F*3,72 = 3.97, *P* = 0.01, Wilks λ = 0.86, η^2^ = 0.14). Successful birds were heavier than unsuccessful birds (successful, 969.5 ± 5.7 g; unsuccessful, 942.0 ± 8.1 g, *F*1,75 = 9.78, *P* = 0.003, *η*^2^ = 0.11). CORT, BUTY and haematocrit levels (separate analyses) did not differ for successful and unsuccessful murres. CORT levels were lower for murres that had more successful breeding attempts in their capture year and in the following three years (so a maximum of 4 chicks fledged), compared to murres with fewer successful attempts (*F*2,42 = 5.20, *P* = 0.01, 3 or 4 fledglings, 14.5 ng/ml, CI 11.8, 17.8; 2 fledglings, 20.4 ng/ml, CI 18.2, 22.9; 1 fledgling, 18.6 ng/ml, CI 15.8, 21.4).

### Sex differences

The overall analysis of sex differences was significant (MANOVA, *F*3,63 = 3.04, *P* = 0.04, Wilks λ = 0.87, η^2^ = 0.13, with Julian capture date as a covariate). Compared to females, males had higher CORT and higher BUTY levels. There were no sex differences in mass, haematocrit levels, or chick-feeding rates (Table 2).

**Table 2:** Means (lower and upper 95% confidence limits) for physiological measures in males and females including *F* values, probability and effect sizes (*η*^2^) for significant variables

Measures	Males	Females	*F* value (df, *P*, *η*^2^)
Mass (g)	967.4 (953.1, 981.7)	954.9 (941.8, 967.9)	Ns
BUTY (mmol/l)	0.98 (0.84, 1.16)	0.82 (0.74, 0.91)	4.25 (1,68, 0.043, 0.06)
CORT (ng/ml)	18.2 (16.6, 20.4)	15.5 (14.3, 17.40)	4.39 (1,68, 0.004, 0.06)
Haematocrit (%)	52.3 (50.8, 53.7)	50.9 (49.4, 52.4)	Ns

### Effects of capture date (proxy for change in indicators during chick rearing)

The overall MANOVA comparing murres captured in the first half of mid-chick rearing (July 8–13) and the second half (July 14–19) was significant (*F*3,78 = 4.92, *P* = 0.004, Wilks *λ* = 0.84, *η*^2^ = 0.16). CORT levels were lower later in the capture period compared to earlier. In contrast, the opposite was the case for BUTY levels, with levels being higher for birds caught later in mid-chick rearing compared to earlier. Neither mass nor haematocrit levels differed with capture date (Table 3).

**Table 3:** Means (lower and upper 95% confidence limits) for physiological measures for murres caught early and late in chick rearing) including *F* values, probability and effect sizes (*η*^2^) for significant variables

Measures	Early	Late	*F* value (df, *P*, *η*^2^)
Mass (g)	961.3 (946.6, 975.9)	959.2 (947.5, 969.3)	Ns
BUTY (mmol/l)	0.78 (0.69, 0.87)	0.99 (0.87, 1.11)	9.93 (1,80, 0.002, 0.11)
CORT (ng/ml)	18.6 (17.0, 20.4)	15.5 (14.1, 16.6)	5.26 (1,80, 0.024, 0.06)
Haematocrit (%)	50.6 (47.3, 53.9)	52.3 (50.9, 53.2)	Ns

## Discussion

Murres were heavier in good years compared to intermediate and poor foraging years. Mass did not vary with capture date, suggesting that mass had already stabilized by the time murres were captured in mid-chick rearing. This observation fits with findings that breeding murres lose body mass right after their chicks hatch (common murres, Wilhelm, 2004; thick-billed murres, Croll *et al.*, 1991; Gaston and Perin, 1993; Elliott *et al.*, 2008), with mass stabilizing later in chick rearing. Successful breeders were heavier than unsuccessful ones. Thick-billed murres that lost the most mass from incubation to chick rearing showed the greatest increase in diving depth (Elliott *et al.*, 2008), suggesting that the lower metabolic rate associated with reduced mass allowed murres to dive longer or deeper. In contrast, Paredes *et al.* (2015) found that heavier thick-billed murres had deeper dives than lighter ones, with the heaviest and lightest murres foraging on different fish species. Thus, it appears that the range of depths that specific fish species inhabit may influence optimal body mass in murres. In addition to adjusting mass to respond to yearly variation in capelin availability, dropping more mass in less optimal years, and elevating haematocrit levels, may be related to increased foraging effort. Table 4 summarizes our findings, using the changes we observed during mid-chick rearing along with findings from earlier literature, to suggest the likely sequence of events in early chick rearing. Taken together, our results and the previous literature suggest that indicators change within breeding phases and so researchers should take care to make timing of sampling consistent until these changes have been documented and understood.

**Table 4:** Physiological changes in mid-chick rearing with inferences about early chick rearing from current results and previous studies (early chick rearing relative to incubation and mid-chick rearing)

Measure	Early chick rearing	Mid-chick rearing	Rationale
**Poor years**			
CORT	Elevated	Decreased	1,2,3; then reduced in response to low foraging success
Mass	Decrease	Stable	4,5; mass loss completed early
BUTY	Elevated	Decreased	6; mass loss completed early
**Intermediate years**			
CORT	Elevated	Elevated	1,3; extra foraging effort may pay off
Mass	Decrease	Stable	4,5; mass loss completed before sampling onset
BUTY	Elevated	Decreased	6;
**Good years**			
CORT	Elevated?	Low	1,2,3; elevation unnecessary
Mass	Some decrease	Decrease may continue	4,5; higher mass than in other year type
BUTY	Elevated	Still elevated	Gradual mass loss ongoing in heavier birds

^1^Doody *et al.* (2008); ^2^Jacobs *et al.* (2013); ^3^Barrett *et al.* (2015); ^4^Croll *et al.* (1991); ^5^Gaston and Hipfner (2006b); ^6^Cerasale and Guglielmo (2006).

BUTY levels were higher in good years than in poor and intermediate years. BUTY levels may be an indicator of both the timing and extent of mass loss in murres. Thus, by the time we captured the adults in mid-chick rearing, adults with low BUTY levels had presumably already lost their maximal amount of breeding-associated mass, losses that occur earlier in a poor year (Wilhelm, 2004). Good years were an exception, where higher BUTY levels may mean that birds could delay mass loss so that it was still ongoing when birds were captured.

The relationship between foraging conditions and CORT levels was not simply the reverse of the relationship to mass. Murres had higher CORT levels in the intermediate years than either the poor or good years, producing an inverted U-shaped relationship. The higher CORT baseline levels in intermediate years (10% higher than in good years; 22% higher than in poor years) likely involve both the high-affinity mineralocorticoid (MR)/Type I and the low-affinity glucocorticoid (GR)/Type II receptors (Romero, 2004; Landys *et al.*, 2006; Breuner, 2010). Stimulation of Type I receptors is related to increased foraging effort, favouring energy storage and chick feeding, whereas stimulation of Type II receptors may induce metabolism of body components, including lipids (Landy*s et al.*, 2006). We see evidence of both here with higher CORT levels in intermediate years (likely promoting chick feeding) and lower CORT levels in birds with the highest number of fledged chicks (likely reflecting a balance between parental investment and self-maintenance). How increased CORT affects individuals should depend on their condition and recent additional stressors that determine whether they exceed their personal thresholds for transitioning from normal ‘reactive’ into ‘emergency homoeostasis’ (as per Romero *et al.*, 2009).

Long-lived slow-pace-of-life species, such as seabirds, should show a greater CORT response to environmental stressors than fast-pace-of-life species that have shorter lifespans with fewer breeding seasons (Breuner, 2010). Thus, for the former, individuals should mount a CORT increase in response to difficult foraging conditions (Bókony *et al.*, 2009), with chicks being fed more or less depending on parental quality (Angelier *et al.*, 2007; Doody *et al.*, 2008; Crossin *et al.*, 2012). CORT elevations may be most beneficial when foraging conditions are not optimal (intermediate years), but are good enough that extra effort will result in higher foraging returns. In contrast, CORT levels in murres may be lower both in good years, when less effort is required, and in poor years when increased effort is unlikely to pay off and sustained high CORT levels would be deleterious.

The decrease in CORT levels as chick rearing progressed may have prevented mass loss exceeding a prudent threshold or, alternatively, the decrease may reflect short-term changes in food availability. Barrett *et al.* (2015) found that decreasing CORT levels in common murres closely tracked increased abundance of larval cod. Thus, the decrease in CORT levels in our sample may be due to a general improvement in feeding conditions as the chick-rearing period progressed. Indeed, the lack of CORT decrease in this study in two out of four intermediate years in which capelin availability decreased later in chick rearing is consistent with Barrett *et al.* (2015).

Previous research on common murres has suggested a linear relationship between elevated CORT levels and low food abundance (Kitaysky *et al.*, 2007; Doody *et al.*, 2008; Barrett *et al.*, 2015), results that apparently contradict our current results. However, the ‘mismatch’ capelin year in Doody *et al.* (2008) was not nearly as bad as the years we have classified as ‘poor’ in the current study (same capelin diary information and spawning records were used in both studies). Thus, the appropriate comparison is between the intermediate years in the current study and the mismatch year in the Doody *et al.* (2008) study. In both studies, CORT was elevated in years with lower food abundance, with very similar absolute CORT levels, at a time when increased effort should have resulted in increased food acquisition. Having this range of years of different capelin availabilities in a long-term study may help to clarify some of the issues about the relationship between CORT and feeding found in the literature: a ‘poor’ year simply may be just the worst year in a particular study. How bad this ‘poor’ year is, relative to the typical range for that species, may differ across studies (e.g. Doody *et al.*, 2008 vs. the current study). Long-term studies also make it possible to contrast physiological indicators associated with current versus longer-term reproductive success. Thus, while birds had *higher* CORT levels in intermediate compared to other years, supporting the CORT-adaptation hypothesis, murres with greater longer-term (current plus the next three years) success had *lower* CORT levels (see Angelier *et al.*, 2010), supporting the CORT-fitness hypothesis. Research on the ancient murrelets (*Synthliboramphus antiquus*), another alcid species also supports the CORT-fitness hypothesis: birds incubating a single egg, with presumably higher pre-lay stress, had higher CORT levels than birds with two eggs (Shoji *et al.*, 2013).

Males had higher CORT and BUTY levels than females but they did not have higher chick-feeding rates. These results suggest that males and female murres in this colony differ in their physiology but not obviously in their behaviour (see also Jacobs *et al.* 2013; Takahashi *et al.*, 2017). The differences in physiological indicators may be related to their different post-fledging roles; males alone take the chicks to sea while females can remain in the colony for some time after the male and chick have departed. Males may have been selected to delay mass loss in chick rearing so that they generally maintain body mass for the period where they will be feeding both themselves and the chick. Thus, the processes of mass reduction involving increased CORT and BUTY levels, hypothesized here to be generally occurring before we captured parents in mid-chick rearing, may be slightly delayed in males.

Mean haematocrit values (52.1%) were similar to those in previous studies of common (54.8%, Wanless *et al.*, 1997) and thick-billed murres (52.8%, Croll *et al.*, 1992). Murres with lower body mass had higher haematocrit values than heavier murres, a finding that differs from results from many other species (as in Fair *et al.*, 2007). Further, murres had higher haematocrits in poor years than in good ones. One strategy of murres foraging near Gull Island is to dive deeply enough (>50 m) to reach the cold intermediate level where capelin swim less quickly (Hedd *et al.*, 2009). Murres that lost the most mass dove deeper than murres that lost less mass (Elliott *et al.*, 2008). Taken together, these results and previous studies add weight to the suggestion that when fish are less available, lighter birds with higher haematocrit levels can make more frequent, deeper or longer dives than heavier birds with lower haematocrit levels (as in Crossin *et al.*, 2015).

Recent changes in ocean temperature have affected the forage species that murres and other seabird species catch to feed to their chicks (e.g. Franci *et al.*, 2015). So far, impacts in the western North Atlantic have been year, area and species-specific. For example, 2012, the year that northern gannets *Morus bassanus* temporarily abandoned the breeding colonies in eastern North America was associated with unusually high sea surface temperatures (Montevecchi *et al.*, 2013; Franci *et al.*, 2015). Despite the serious impact on gannet productivity, 2012 was a good year for murres (current study) and Atlantic puffins *Fratercula arctica* (Fitzsimmons *et al.*, 2017) in eastern Newfoundland colonies. In contrast, cold temperatures and storms that drove capelin off shore late in the 2011 breeding season in eastern Newfoundland had minimal impact on murres (an intermediate year in this study), but had a severe impact on mortality in the later-fledging Atlantic puffin chicks (Fitzsimmons *et al.*, 2017). Despite a few encouraging reports of foraging flexibility (e.g. Bryant *et al.*, 1999, common and thick-billed murres), further declines in forage fish, due to increasing sea surface temperatures that affect spawning decisions and/or availability of their prey, will seriously affect seabird productivity (reviewed in Grémillet and Boulinier, 2009). A better understanding of the physiological impact of changes in fish availability may help the scientific community understand the reasons for productivity declines in shorter time scales than population changes. It is also possible that these results could be applied to species with a longer chick-rearing period, such as Atlantic puffins, where measures of mass, haematocrit, and BUTY measured in the field with a ketone reader could be used to predict the condition of the fish stocks throughout most of the breeding season.

Are murres behaving as prudent parents or do they take on extra mortality or future fecundity risk in order to succeed in the current breeding attempt? Stabilization of mass and the decreased CORT levels by mid-chick rearing appear prudent, with murres adjusting their investment to fit with particular foraging conditions. We found, however, that successful, possibly high quality, murres differed from other birds in several ways: higher body mass, and lower CORT, associated with current nest success or long-term nest success, respectively, suggesting that some individuals can better withstand the exercise requirements of chick rearing (Yap *et al.*, 2017). These results support the suggestion that we should examine a variety of physiological indicators to better understand breeding costs (Williams and Fowler, 2015) and that we should consider that the timing of sampling within breeding periods may affect the conclusions we reach. Although some results in this study support a prudent parent model, it is important to note that apparently excessive costs in the short term (such as elevated CORT in intermediate years) should be viewed in terms of long-term stochastic variation in resources, over which selection has operated to shape reproductive decisions (Erikstad *et al.*, 1997; Satterthwaite *et al.*, 2010).

## References

[cox055C1] Ainley D, Nettleship D, Carter H, Storey AE (2002) Common murres (*Uria aalge*) In Poole A (ed) The Birds of North America Online. Cornell Lab of Ornithology, Ithaca http://bna.birds.cornell.edu/bna/species/666 doi:10.2173/bna.666.

[cox055C2] Almasi B, Roulin A, Jenni-Eiermann S, Jenni L (2008) Parental investment and its sensitivity to corticosterone is linked to melanin-based coloration in barn owls. Horm Behav 54:217–223. 18413149 10.1016/j.yhbeh.2008.02.021

[cox055C3] Angelier F, Clément-Chastel C, Gabrielsen GW, Chastel O (2007) Corticosterone and time–activity budget: an experiment with Black-legged kittiwakes. Horm Behav 52:482–491. 17707380 10.1016/j.yhbeh.2007.07.003

[cox055C4] Angelier F, Bost C-A, Giraudeau M, Bouteloup G, Dano S, Chastel O (2008) Corticosterone and foraging behavior in a diving seabird: the Adélie penguin, *Pygoscelis adeliae*. Gen Comp Endocr 156:134–144. 18221738 10.1016/j.ygcen.2007.12.001

[cox055C5] Angelier F, Wingfield JC, Weimerskirch H, Chastel O (2010) Hormonal correlates of individual quality in a long-lived bird: a test of the ‘corticosterone–fitness hypothesis’. Biol Lett 6:846–849. 20573614 10.1098/rsbl.2010.0376 PMC3001372

[cox055C6] Barrett RT, Erikstad KE, Sandvik H, Myksvoll M, Jenni-Eiermann S, Kristensen DL, Moum T, Reiertsen TK, Vikebø F (2015) The stress hormone corticosterone in a marine top predator reflects short-term changes in food availability. Ecol Evol 5:1306–1317. 25859335 10.1002/ece3.1438 PMC4377273

[cox055C7] Blem CR (1976) Patterns of lipid storage and utilization in birds. Am Zool 16:67 l–684.

[cox055C8] Bókony V, Lendvai AZ, Liker A, Angelier F, Wingfield JC, Chastel O (2009) Stress response and the value of reproduction: are birds prudent parents? Am Nat 173:589–598. 19281425 10.1086/597610

[cox055C9] Bonier F, Moore IT, Martin PR, Robertson RJ (2009) The relationship between fitness and baseline glucocorticoids in apasserine bird. Gen Comp Endocr 163:208–213. 19135999 10.1016/j.ygcen.2008.12.013

[cox055C10] Bonier F, Moore IT, Robertson RJ (2011) The stress of parenthood? Increased glucocorticoids in birds with experimentally enlarged broods. Biol Lett 7:944–946. 21632615 10.1098/rsbl.2011.0391 PMC3210667

[cox055C11] Breuner C (2010) Stress and reproduction in birds In Norris D (ed) Hormones and Reproduction in Vertebrates. Elsevier, Oxford.

[cox055C12] Bryant R, Jones IL, Hipfner JM (1999) Responses to changes in prey availability by Common Murres and Thick-billed Murres at the Gannet Islands, Labrador. Can J Zool 77:1278–1287.

[cox055C13] Buren AD, Koen-Alonso M, Pepin P, Mowbray F, Nakashima B, Stenson G, Ollerhead N, Montevecchi WA (2014) Bottom-up regulation of capelin, a keystone forage species. PLoS ONE 9:1–11. 10.1371/journal.pone.0087589 PMC3913657 24503909

[cox055C14] Burger AE, Piatt JF (1990) Flexible time budgets in breeding Common Murres: buffers against variable prey abundance. Stud Avian Biol 14:71–83.

[cox055C15] Cerasale DJ, Guglielmo CG (2006) Dietary effects on prediction of body mass changes in birds by plasma metabolites. Auk 123:836–846.

[cox055C16] Carscadden J, Nakashima BS, Frank KT (1997) Effects of fish length and temperature on the timing of peak spawning in capelin (*Mallotus villosus*). Can J Fish Aquat Sci 54:781–787.

[cox055C17] Cherel Y, Le Mayo Y (1985) Five months of fasting in king penguin chicks: body mass loss and fuel metabolism. Am J Physiol 18:R387–R392. 10.1152/ajpregu.1985.249.4.R387 4051024

[cox055C18] Cherel Y, Le Mayo Y (1988) Changes in body mass and plasma metabolites during short-term fasting in the king penguin. Condor 90:257–258.

[cox055C19] Crespi EJ, Williams TD, Jessop TS, Delahanty B (2013) Life history and the ecology of stress: how do glucocorticoid hormones influence life-history variation in animals? Funct Ecol 27:93–106.

[cox055C20] Croll DA, Gaston AJ, Noble DG (1991) Adaptive loss of mass in thick-billed murres. Condor 93:496–502.

[cox055C21] Croll DA, Gaston AJ, Burger AE, Konnoff D (1992) Foraging behavior and physiological adaptation for diving in thick-billed murres. Ecology 73:344–356.

[cox055C22] Crossin GT, Takahashi A, Sakamoto KQ, Trathan PN, Williams TD (2015) Habitat selection by foraging macaroni penguins correlates with hematocrit, an index of aerobic condition. Mar Ecol Prog Ser 530:163–176.

[cox055C23] Crossin GT, Trathan PN, Phillips RA, Gorman KB, Dawson A, Sakamoto KQ, Williams TD (2012) Corticosterone predicts foraging and parental care in macaroni penguins. Am Nat 180:31–41. 10.1086/666001 22673661

[cox055C24] Dawson RD, Bortolotti GR (1997) Are avian hematocrits indicative of condition? American kestrel as a model. J Wildlife Manage 61:1297–1306.

[cox055C25] Doody LM, Wilhelm SI, McKay DW, Walsh CJ, Storey AE (2008) The effects of variable foraging conditions on common murre (*Uria aalge*) corticosterone concentrations and parental provisioning. Horm Behav 53:140–148. 17991465 10.1016/j.yhbeh.2007.09.009

[cox055C26] DFO (2015) Proceedings of the regional peer review meeting of the framework for Atlantic herring (*Clupea harengus*) and reference points for Capelin (*Mallotus villosus*) in the Newfoundland and Labrador Region; November 19–21, 2013. DFO Can Sci Advis Sec Proceed Ser 2014/049.

[cox055C27] Drent RH, Daan S (1980) The prudent parent: energetic adjustments in avian breeding. Ardea 68:225–252.

[cox055C28] Elliott KH, Jacobs SR, Ringrose J, Gaston AJ, Davoren GK (2008) Is mass loss in Brünnich’s guillemots *Uria lomvia* an adaptation for improved flight performance or improved dive performance? J Avian Biol 39:619–628.

[cox055C29] Elliott KH, Ricklef RE, Gaston AJ, Hatch SA, Speakman JR, Davoren GK (2013) High flight costs, but low dive costs, in auks support the biomechanical hypothesis for flightlessness in penguins. Proc Natl Acad Sci USA 110:9380–9384. 23690614 10.1073/pnas.1304838110 PMC3677478

[cox055C30] Elliott KH, Shoji A, Campbell KL, Gaston AJ (2010) Oxygen stores and foraging behavior of two sympatric, planktivorous alcids. Aquat Biol 8:221–235.

[cox055C32] Erikstad KE, Asheim M, Fauchald P, Dahlburg L, Tyeraa T (1997) Adjustment of parental effort in the puffin: the role of adult body condition and chick size. Behav Ecol Sociobiol 40:95–100.

[cox055C33] Fair J, Whitaker S, Pearson B (2007) Sources of variation in haematocrit in birds. Ibis 149:535–552.

[cox055C34] Fitzsimmons MG, Rector ME, McKay DW, Storey AE (2017) High growth and low corticosterone in food-supplemented Atlantic puffin *Fratercula arctica* chicks under poor foraging conditions. Mar Ecol Prog Ser 565:217–226.

[cox055C35] Franci CD, Vézina F, Grégoire F, Rail J-F, Verreault J (2015) Nutritional stress in Northern gannets during an unprecedented low reproductive success year: can extreme sea surface temperature event and dietary change be the cause? Comp Biochem Physiol A 181:1–6. 10.1016/j.cbpa.2014.11.017 25449633

[cox055C36] Fridolfsson A-K, Ellegren H (1999) A simple and universal method for molecular sexing of non-ratite birds. J Avian Biol 30:116–121.

[cox055C37] Gaston AJ, Perin S (1993) Loss of mass in breeding Brünnich’s guillemots *Uria lomvia* is triggered by hatching. Ibis 135:472–474.

[cox055C38] Gaston AJ, Hipfner JM (2006a) Adult Brünnich’s guillemots *Uria lomvia* balance body condition and investment in chick growth. Ibis 148:106–113.

[cox055C39] Gaston AJ, Hipfner JM (2006b) Body mass changes in Brünnich’s Brunnich’s guillemots *Uria lomvia* with age and breeding stage. J Avian Biol 37:101–109.

[cox055C40] Golet GH, Schmutz JA, Irons DB, Estes JA (2004) Determinants of reproductive costs in the long-lived black-kittiwake: a multiyear experiment. Ecol Monogr 74:353–372.

[cox055C41] Grémillet D, Boulinier T (2009) Spatial ecology and conservation of seabirds facing global climate change: a review. Mar Ecol Prog Ser 391:121–137.

[cox055C42] Groscolas R, Robin J-P (2001) Long-term fasting and re-feeding in penguins. Comp Biochem Physiol A Mol Integr Physiol A 128:645–655. 10.1016/s1095-6433(00)00341-x 11246051

[cox055C43] Guglielmo CG, O’Hara PD, Williams TD (2002) Extrinsic and intrinsic sources of variation in plasma lipid metabolites of free-living western sandpipers (*Calidris mauri*). Auk 119:437–445.

[cox055C44] Guglielmo CG, Cerasale DJ, Eldermire C (2005) A field validation of plasma metabolite profiling to assess refueling performance of migratory birds. Physiol Biochem Zool 78:116–125. 15702470 10.1086/425198

[cox055C45] Harding AMA, Piatt JF, Schmutz JA, Shultz MT, Van Pelt TI, Kettle AB, Speckman SG (2007) Prey density and the behavioral flexibility of a marine predator: the common Murre (*Uria aalge*). Ecology 88:2024–2033. 17824434 10.1890/06-1695.1

[cox055C46] Harris MP, Wanless S (1988) The breeding biology of guillemots Uria aalge on the Isle of May over a six year period. Ibis 130:172–192.

[cox055C47] Hedd A, Regular PM, Montevecchi WA, Buren AD, Burke CM, Fifield DA (2009) Going deep: common murres dive into frigid water for aggregated, persistent and slow-moving capelin. Mar Biol 156:741–751.

[cox055C48] Hedrick MS, Duffield DA (1991) Hematological and rheological characteristics of blood in seven marine mammal species: physiological implications for diving behavior. J Zool 225:273–283.

[cox055C49] Horton BM, Holberton RL (2009) Corticosterone manipulations alter morph-specific nestling provisioning behavior in male white-throated sparrows, *Zonotrichia albicollis*. Horm Behav 56:510–518. 19751738 10.1016/j.yhbeh.2009.09.001

[cox055C50] Jacobs SR, Edwards DB, Ringrose J, Elliot KH (2011) Changes in body composition during breeding: reproductive strategies of three species of seabirds under poor environmental conditions. Comp Biochem Physiol B 158:77–82. 20888927 10.1016/j.cbpb.2010.09.011

[cox055C100] Jacobs SR, Elliott KH, Gaston AJ (2013) Parents are a drag: long-lived birds share the cost of increased foraging effort with their offspring, but males pass on more of the costs than females. PLoS ONE 8(1):e54594. 23382921 10.1371/journal.pone.0054594 PMC3559872

[cox055C51] Jacobs SR, Elliott KH, Guigueno MF, Gaston AJ, Redman P, Speakman JR, Weber JM (2012) Determining seabird body condition using nonlethal measures. Physiol Biochem Zool 85:85–95. 22237292 10.1086/663832

[cox055C52] Jones IL (1994) Mass changes of least auklets *Aethia pusilla* during the breeding season: evidence for the programmed loss of mass. J Anim Ecol 63:71–78.

[cox055C53] Kitaysky AS, Piatt JF, Wingfield JC (2007) Stress hormones link food availability and population processes in seabirds. Mar Ecol Prog Ser 352:245–258.

[cox055C54] Landys MM, Ramenofsky M, Wingfield JC (2006) Actions of glucocorticoids at a seasonal baseline as compared to stress-related levels in the regulation of periodic life processes. Gen Comp Endocr 148:132–149. 16624311 10.1016/j.ygcen.2006.02.013

[cox055C55] Montevecchi WA, Chardine J, Rail J-F, Garthe S, Pelletier D, Regular P, Burke C, Hedd A, McFarlane Tranquilla L, Bennett S, et al (2013) Extreme event in a changing ocean climate: warm-water perturbation of 2012 influences breeding gannets and other marine animals in the Northwest Atlantic. Osprey 44:14–19.

[cox055C56] Mowbray FK (2014) Recent spring offshore acoustic survey results for capelin, *Mallotus villosus*, in NAFO Division 3 L. DFO Can Sci Advis Sec Res Doc 2013/040. v + 25 p.

[cox055C57] Nakashima BS (1996) The relationship between oceanographic conditions in the 1990 s and changes in spawning behaviour, growth and early life history of capelin (*Mallotus villosus*). NAFO Sci Coun Studies 24:55–68.

[cox055C58] Niizuma Y, Araki Y, Mori H, Takahashi A, Watanuki Y (2002) Responses of body components to changes in the energetic demand throughout the breeding stages of rhinoceros auklets. Can J Zool 80:1549–1555.

[cox055C59] Paredes R, Orben RA, Roby DD, Irons DB, Young R, Renner H, Tremblay Y, Will A, Harding AM, Kitaysky AS (2015) Foraging ecology during nesting influences body size in a pursuit-diving seabird. Mar Ecol Prog Ser 533:261–276.

[cox055C60] Piatt JF, Harding AMA, Shultz M, Speckman SG, van Pelt TI, Drew GS, Kettle AB (2007) Seabirds as indicators of marine food supplies: Cairns revisited. Mar Ecol Prog Ser 352:221–234.

[cox055C61] Rector ME, Kouwenberg A-L, Wilhelm SI, Robertson GJ, McKay DW, Fitzsimmons MG, Baker CR, Cameron-MacMillan ML, Walsh CJ, Storey AE (2012) Corticosterone levels of Atlantic puffins vary with breeding stage and sex but are not elevated in poor foraging years. Gen Comp Endocr 178:408–416. 22732081 10.1016/j.ygcen.2012.06.008

[cox055C62] Regular PM, Hedd A, Montevecchi WA, Robertson GJ, Storey AE, Walsh CJ (2014) Why timing is everything: energetic costs and reproductive consequences of resource mismatch for a chick-rearing seabird. Ecosphere 5:1–13.

[cox055C63] Robin J-P, Frain M, Sardet C, Groscolas R, Le Maho Y (1988) Protein and lipid utilization during long-term fasting in emperor penguins. Am J Physiol 254:R61–R68. 3337270 10.1152/ajpregu.1988.254.1.R61

[cox055C64] Romero LM (2004) Physiological stress in ecology: lessons from biomedical research. Trends Ecol Evol 19:249–255. 16701264 10.1016/j.tree.2004.03.008

[cox055C65] Romero LM, Dickens MJ, Cyr NE (2009) The reactive scope model—a new model integrating homeostasis, allostasis, and stress. Horm Behav 55:375–389. 19470371 10.1016/j.yhbeh.2008.12.009

[cox055C66] Sagone AL, Lawrence T, Balcerzak SP (1973) Effect of smoking on tissue oxygen supply. Blood 41:845–851. 4712210

[cox055C67] Satterthwaite WH, Kitaysky AS, Hatch SA, Piatt JF, Mangel M (2010) Unifying quantitative life-history theory and field endocrinology to assess prudent parenthood in a long-lived seabird. Evol Ecol Res 12:779–792.

[cox055C68] Seewagen CL, Sheppard CD, Slayton EJ, Guglielmo CG (2011) Plasma metabolites and mass changes of migratory landbirds indicate adequate stopover refueling in a heavily urbanized landscape. Condor 113:284–297.

[cox055C69] Shoji A, Elliott KH, O’Reilly KM, Gaston AJ (2013) High corticosterone, not high energy cost, correlates with reproductive success in the burrow-nesting ancient murrelet. PLoS ONE 8(12): e84280. 24391929 10.1371/journal.pone.0084280 PMC3877242

[cox055C71] Stearns SC (1989) Trade-offs in life-history evolution. Funct Ecol 3:259–268.

[cox055C72] Takahashi LS, Storey AE, Wilhelm SI, Walsh CJ (2017) Turn-taking ceremonies in a colonial seabird: does behavioral variation signal individual condition? Auk 134:530–541.

[cox055C73] Thornton SJ, Hochachka PW (2004) Oxygen and the diving seal. Undersea Hyperb Med 31:81–95. 15233163

[cox055C74] Totzke U, Fenske M, Hüppop O, Raabe H, Schach N (1999) The influence of fasting on blood and plasma composition of herring gulls (*Larus argentatus*). Physiol Biochem Zool 72:426–437. 10438680 10.1086/316675

[cox055C75] Vleck CM, Vertalino N, Vleck D, Bucher TL (2000) Stress, corticosterone, and heterophil to lymphocyte ratios in free-living Adélie Penguins. Condor 102:392–400.

[cox055C76] Wanless S, Barton TR, Harris MP (1997) Blood hematocrit measurements of 4 species of North Atlantic seabirds in relation to levels of infestation by the tick *Ixodes uriae*. Colon Waterbirds 20:540–544.

[cox055C77] Wilhelm SI (2004) Behavioral and physiological responses of breeding common Murres (*Uria aalge*): exploring inter-annual variability within individuals. Doctoral dissertation, Memorial University.

[cox055C78] Wilhelm SI, Walsh CJ, Storey AE (2008) Time budgets of Common Murres vary in relation to changes in inshore capelin availability. Condor 110:316–324.

[cox055C79] Williams CT, Kitaysky AS, Kettle AB, Buck L (2008) Corticosterone levels of tufted puffins vary with breeding stage, body condition index, and reproductive performance. Gen Comp Endocr 158:29–35. 18547575 10.1016/j.ygcen.2008.04.018

[cox055C80] Williams TD, Fowler MA (2015) Individual variation in workload during parental care: can we detect a physiological signature of quality or cost of reproduction? J Ornithol 156:S441–S451.

[cox055C81] Yap KN, Serota MW, Williams TD (2017) The physiology of exercise in free-living vertebrates: what can we learn from current model systems? Integrative Comp Biol 1–12. doi:10.1093/icb/icx016. 10.1093/icb/icx016 28662569

